# Functional attributes of epilithic diatoms for palaeoenvironmental interpretations in South-West Greenland lakes

**DOI:** 10.1007/s10933-017-9968-9

**Published:** 2017-06-09

**Authors:** Suzanne McGowan, Hazel V. Gunn, Erika J. Whiteford, N. John Anderson, Vivienne J. Jones, Antonia C. Law

**Affiliations:** 1 School of Geography, University of Nottingham, University Park, Nottingham, NG7 2RD UK; 2 School of Geography, University of Nottingham, Malaysia Campus, 43500 Semenyih, Selangor Darul Ehsan Malaysia; 3 Department of Geography, Loughborough University, Loughborough, LE11 3TU Leicestershire UK; 4 Environmental Change Research Centre, Department of Geography, University College London, Gower Street, London, WC1E 6BT UK; 5 School of Physical and Geographical Sciences, Keele University, William Smith Building, Keele, Staffs ST55BG UK

**Keywords:** Arctic, Biofilm, Climate, Taphonomy, Nitrogen-limitation, Seasons, Snowmelt

## Abstract

Benthic diatoms are commonly used for palaeoenvironmental reconstruction in Arctic regions, but interpretation of their ecology remains challenging. We studied epilithic diatom assemblages from the shallow margins of 19 lakes from three areas (coast-inland-ice sheet margin) along a climate gradient in Kangerlussuaq, West Greenland during two periods; shortly after ice-off (spring) and in the middle of the growth season (summer). We aimed to understand the distribution of Arctic epilithic diatoms in relation to water chemistry gradients during the two seasons, to investigate their incorporation into lake sediments and to assess their applicability as palaeoenvironmental indicators. Diatoms were correlated with nutrients in the spring and alkalinity/major ions in the summer, when nutrients were depleted; approximately half of the variance explained was independent of spatial factors. When categorised by functional attributes, diatom seasonal succession differed among regions with the most obvious changes in inland lakes where summer temperatures are warmer, organic nutrient processing is prevalent and silicate is limiting. These conditions led to small, motile and adnate diatoms being abundant in inland lakes during the summer (*Nitzschia* spp., *Encyonopsis microcephala*), as these functional attributes are suited to living within complex mats of non-siliceous microbial biofilms. Seasonal succession in silica-rich lakes at the coast was less pronounced and assemblages included *Tabellaria flocculosa* (indicating more acidic conditions) and *Hannaea arcus* (indicating input from inflowing rivers). The nitrogen-fixing diatom *Epithemia sorex* increased from the coast to the ice sheet, negatively correlating with a gradient of reactive nitrogen. The presence of this diatom in Holocene sediment records alongside cyanobacterial carotenoids during arid periods of low nitrogen delivery, suggests that it is a useful indicator of nitrogen limitation. *Nitzschia* species appear to be associated with high concentrations of organic carbon and heterotrophy, but their poor representation in West Greenland lake sediments due to taphonomic processes limits their palaeoenvironmental application in this region. Proportions of epilithic taxa in lake sediment records of coastal lakes increased during some wetter periods of the Holocene, suggesting that snowpack-derived nutrient delivery may offer diatom taxa living at lake margins a competitive advantage over planktonic diatoms during the “moating” ice melt period. Thus, further research investigating linkages between epilithic diatoms, snowpack and nutrient delivery in seasonally frozen lakes is recommended as these taxa live on the ‘front-line’ during the spring and may be especially sensitive to changes in snowmelt conditions.

## Introduction

Diatom assemblages in lake sediments are commonly used as palaeoenvironmental indicators in Arctic regions (Bradley et al. 1996; McGowan et al. 2003; Jones and Birks 2004; Smol et al. 2005; Wolfe et al. 2006; Perren et al. 2012). Effective interpretation of diatom records requires ecological information which is commonly obtained via surface sediment “training sets”, to determine correlations among diatoms and physical/chemical properties of lakes and define optimum and tolerance ranges for individual taxa (Ryves et al. 2002; Antoniades et al. 2005a; Michelutti et al. 2006; Lim et al. 2001a; Michelutti et al. 2007a). Extracting meaningful ecological information from Arctic training sets is challenging because environmental gradients are generally short, with low concentrations of nutrients and major ions. Training sets that span ecotonal boundaries usually record greater variability in physicochemical properties which correlate with catchment vegetation, but strong co-variability among lake variables limits the ecological information that can be extracted (Bouchard et al. 2004; Lim et al. 2007; Juggins 2013). Most primary production in Arctic lakes occurs in benthic areas (Vadeboncoeur et al. 2003) and so training sets which correlate pelagic measurements with diatom assemblages have resulted in poorly defined ecological preferences for many benthic taxa (Antoniades et al. 2005a; Bonilla et al. 2005; Finkelstein and Gajewski 2008).

An alternative “back to basics” method for interpreting diatom records is to use qualitative or semi-quantitative limnological or ecological knowledge of diatoms to interpret assemblage changes. Increases in small *Cyclotella* species are frequently used as indicators of changes in thermal stratification related to warming (Ruhland et al. 2008), because their abundance increases when lake stratification depth decreases (Saros et al. 2016). In High Arctic ponds, changes in the benthic diatom flora after the 18th century are interpreted as a response to warming, with longer ice-free periods expanding the colonisation of mosses and associated diatom epiphytes (Douglas et al. 1994). Such interpretations are more straightforward when environmental changes cause abrupt step changes in lake habitat availability (e.g. stronger stratification, development of aquatic plants). However, in lakes where no thresholds have been crossed and environmental changes are subtle, as is the case in many Arctic sites, this approach has limitations (Michelutti et al. 2007a). Investigating benthic diatoms within individual habitat types can help to define substrate preferences (e.g. sediment biofilms, sediment grains, plants, moss, or rock) and increase the understanding of benthic community structure, enabling greater information to be extracted from the benthic algal record (Michelutti et al. 2003; Pizarro et al. 2004).

Epilithic diatoms grow on rock substrates, and may include associations of attached, motile, epiphytic algae (attached to algal filaments) and facultatively planktonic taxa. Rock substrates are generally inert (but see Blinn et al. 1980) and exposed to a broad range of environmental stressors, and therefore epilithic diatoms are considered to be sensitive bioindicators of environmental conditions (King et al. 2006). Epilithon surveys show that diatoms change across gradients of nutrient (nitrogen, phosphorus) availability (King et al. 2000, 2006) and lake water pH (Cameron et al. 1999; Štefková 2006). Many other factors may influence epilithic diatom assemblages, e.g. invertebrate grazers, wave disturbance, UVR exposure and competition from non-diatom biofilm taxa (Bothwell et al. 1994; Kelly et al. 2009; Miller et al. 1992). However, diatoms which grow at the margins of annually frozen lakes live in “front-line” habitats, the first to receive nutrients and ions (e.g. sulphates) delivered in pulses during snowmelt as lake ice ‘moating’ progresses during the spring (Douglas and Smol 1999; Catalan et al. 2002; Quesada et al. 2008). It seems likely therefore, that littoral diatom assemblages might be highly responsive to the quantity, chemical composition and timing of snow melt. Patterns of Arctic epilithic community succession are not well understood, but it appears that diatom growth peaks early in the growth season (Moore 1974), further highlighting the importance of conditions during the spring. Better understanding of inter-seasonal differences is necessary to fully interpret lake sediment records which integrate diatom production from all seasons.

On death or detachment, epilithic diatoms are transported away from rocks and deposited together with diatoms from other habitats to form part of the lake sediments. The proportion of littoral epilithic diatoms in sediment records is partly dependent on lake bathymetry, which influences the proportion of lake benthos, including stone surfaces, exposed to light (Stone and Fritz 2006). Water level changes also cause major shifts in the proportion of benthic taxa in sediment records, especially in morphometrically uneven lake basins (Anderson and Battarbee 1994; Stevens et al. 2006). Alternatively, changing environmental conditions might shift the relative proportions of diatoms incorporated from different parts of the lake, e.g. some Arctic lakes which are ice covered for longer periods into the summer have proportionally fewer plankton (Keatley et al. 2008). Increases in water clarity associated with anthropogenic acidification or tree line retreat which reduces terrestrial inputs of coloured dissolved organic carbon expand the relative proportion of benthic production (Jones et al. 2011). As well as processes within the lake which alter relative diatom production, modification of the diatom assemblage occurs during and after the deposition in sediments (Ryves et al. 2013). Such taphonomy processes can be influenced by lake physical and chemical characteristics (Ryves et al. 2006), and because epilithic diatoms must be transported considerable distances before deposition in the sedimentary record at the lake centre they might be especially susceptible to damage, dissolution and washout from the lake (Cameron 1995). Despite this complexity, shifts in the abundance of epilithic diatoms are often observed in sedimentary assemblages and the challenge is to better interpret what they mean.

Here we investigated littoral epilithic diatom assemblages in different seasons (spring, summer) in a set of 19 lakes in the Kangerlussuaq area of West Greenland. The lakes span a climate gradient across a transect from the coast to ice sheet margin, and have well studied limnology exhibiting differences in water chemistry, length of ice-free season, snowpack, hydrological connectivity and maximum growth season temperatures. We first used traditional multivariate techniques to explore correlations among epilithic diatoms and water chemistry parameters across the region during each season with partial ordinations to determine whether spatial factors significantly influenced the results. Then, we compared functional attributes of diatoms to understand biofilm community structure differences between spring (early after ice-off) and summer periods. Next, we aimed to determine how well epilithic diatoms were represented in lake sediment records and whether this varied across the lake district. Finally, we tested the application of epilithic diatoms as palaeoenvironmmental indicators in this region where Holocene palaeoclimate and associated environmental changes are relatively well understood by re-visiting some previously published diatom records to determine whether we could extract further information from them.

## Methods

### Site description

**Fig. 1:**
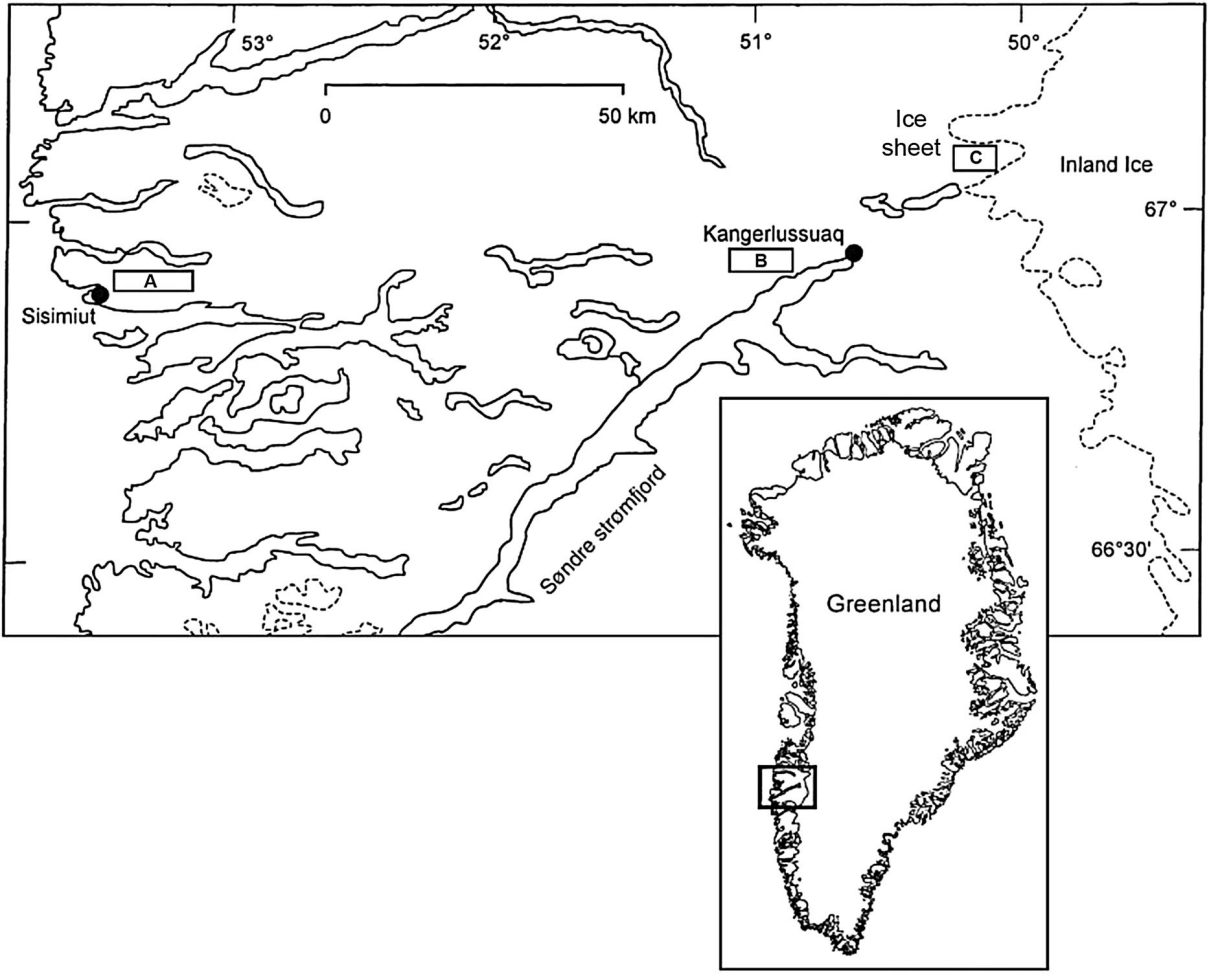
Map of the Kangerlussuaq region revised from Whiteford et al. (2016) showing the location of the three study areas **a** coast, **b** inland and **c** ice sheet margin highlighted in *black boxes* with *dots* representing the closest human settlements

The 180 km transect along Kangerlussuaq is the widest ice- free margin in Greenland, with over 15,000 lakes (Fig. 1) (Anderson and Stedmon 2007). A climate gradient along the transect spans a dry continental Low Arctic climate at the ice sheet margin (annual temperature range of −20 to 10 °C; <150 mm precipitation year^−1^) to increasingly maritime conditions at the coast (annual temperature range of −16 to 6 °C; >500 mm precipitation year^−1^). To investigate changes in biota along this climate gradient, 19 freshwater lakes were selected from three main areas along the transect: (1) the coast (close to the town of Sisimiut but above the marine limit), (2) inland (close to the town of Kangerlussuaq) and (3) the ice sheet margin within a few kilometres of the ice sheet but not directly receiving glacial meltwater (Fig. 1). Ice melt usually occurs at the start of June in the inland lakes, slightly later at the ice sheet lakes (which are cooled by katabatic winds) and more than two–three weeks later at the coast, where snow cover is three times greater and spring temperatures rise more slowly and so lakes may be ‘moated’ for several weeks (Whiteford et al. 2016). Lakes in the coastal area are generally connected to perennial stream networks which flow during the ice-free period whereas lakes in the inland and ice sheet regions are mostly hydrologically isolated because of the scarcity of precipitation. Lakes ranged in depth from 3 to 37 m and area from 4 to 38 ha and were formed mainly by glacial action scraping out basins, resulting in variable morphometries (Table 1).

**Table 1:** Summary of water chemistry and physical properties of study lakes in three areas across south-west Greenland (coast-inland-ice sheet margin) during two sampling occasions (spring, summer)

Area	Lakes sampled	Collection date	Max depth (m)	Lake area (ha)	TP (µg L^−1^)	*SRP (µg L^−1)^	NO_3_–N (µg L^−1)^	NH_4_–N (µg L^−1^)	DIN	TN (µg L^−1^)	SiO_4_ (µg L^−1^)
Summer											
Coast	AT1, AT2, AT4, AT5, AT6, AT7, AT8	1–7 August 2010	11–23	4–12	0–36.7	0–4.5	0	0–2.6	0–2.6	222–572	566–1158
Inland	SS2, SS8, SS85, SS1333, SS1341, SS1381, SS1590	5–12 August 2010	2.9–21	7–37	0–17.4	0–7.1	0	0–8.8	0–8.8	280–970	8.5–580
Ice sheet	SS901, SS902, SS903, SS904, SS905, SS906	24–28 July 2010	11–23	9–38	0–28.8	0–3.6	0	0–6.8	0–6.8	370–758	0–228
Spring											
Coast	AT1, AT2, AT4, AT5, AT6, AT7	1–4 July 2011	11–23	4–12	0–0	0.5–3.9	0–35.8	0–1.6	0–37.5	33–87	0–648
Inland	SS2, SS8, SS85, SS1333	19–23 June 2011	23–19	7–37	0–8.8	0.5–3.7	0	0–10.8	0–10.8	661–1060	219–598
Ice sheet	SS901, SS902, SS903, SS904, SS905, SS909	20–27 June 2011	11–37	9–38	2.2–14.7	1.4–17.8	0	0–1.6	0–1.6	148–469	59–521

Area	Lakes sampled	Collection date	Na^+^ (mg L^−1^)	K^+^ (mg L^−1)^	Mg^2+^ (mg L^−1)^	Ca^2+^ (mg L^−1)^	Cl^−^ (mg L^−1)^	SO_4_^2−^ (mg L^−1)^	Alkal-inity (µeq L^−1)^	NPOC (mg L^−1)^
Summer										
Coast	AT1, AT2, AT4, AT5, AT6, AT7, AT8	1–7 August 2010	2.0–10.5	0.2–5.5	1.4–7.3	2.5–15.2	2.6–5.4	1.5–8.9	200–300	1.1–2.2
Inland	SS2, SS8, SS85, SS1333, SS1341, SS1381, SS1590	5–12 August 2010	15.3–45.8	6.8–20.9	10.0–27.8	16.5–32.7	21.3–70.4	0.9–2.1	5800–13,900	16.3–36.6
Ice sheet	SS901, SS902, SS903, SS904, SS905, SS906	24–28 July 2010	2.0–8.1	1.8–5.8	2.9–10.5	7.5–18.2	2.1–7.2	3.0–5.4	1800–5200	4.5–8.1
Spring										
Coast	AT1, AT2, AT4, AT5, AT6, AT7	1–4 July 2011	1.6–3.3	0.4–0.6	1.7–2.5	5.6–8.4	1.2–3.7	2.1–7.7	100–100	23.5–52.2
Inland	SS2, SS8, SS85, SS1333	19–23 June 2011	18.5–39.0	7.7–16.9	11.9–26.8	26.3–29.6	24.1–55.4	1.2–2.3	2200–3550	16.2–34.9
Ice sheet	SS901, SS902, SS903, SS904, SS905, SS909	20–27 June 2011	2.2–5.9	1.9–5.0	3.2–9.4	8.7–10.9	2.2–5.8	2.8–4.9	550–1400	2.6–9.1

Values given represent ranges for TP = total phosphorus, SRP = soluble reactive phosphorus, DIN = dissolved inorganic nitrogen, TN = total nitrogen, NPOC = non-particulate organic carbon. All variables were log(x + 1) transformed for multivariate analyses except those highlighted with an asterisk (*). Further details on water chemisty of individual lakes are given in Whiteford et al. (2016)

### Sample collection

Epilithic diatoms were collected from rocks in 19 lakes across the three areas during the mid-summer of 2010 (late July to early August) and the period immediately following ice-off in Spring 2011 (late June to early July) (Table 1). Collection dates across the region in spring reflect the order in which the ice melted from each area (inland- ice sheet margin- coast) and samples were collected at coastal lakes whilst remnants of ice remained in the centre of lakes. Diatoms were sampled and combined from five approximately fist-sized (about 100–200 mm diameter) submerged rocks <3 m from the edge at a water depth of around 50 cm, removed using a soft brush and preserved with Lugol’s iodine (King et al. 2006). Diatom slides were prepared following digestion with hydrogen peroxide and mounted using Naphrax resin following Battarbee et al. (2001). A minimum of 300 diatom valves per slide was counted using a Leica DME light microscope at 1000× magnification. Epilithic diatoms were identified with reference to Krammer and Lange-Bertalot (1986–1991) and online resources (Kelly et al. 2005; Spaulding et al. 2010). Each taxon was given a code used in numerical analyses and the most recent nomenclature and authorities are given in Table 2. Diatom counts were converted to percentage relative abundance for further analysis.

**Table 2:** Diatom taxa from the spring and summer epilithon samples from (>2% abundance in the assemblage) and codes used in the manuscript with recent taxonomic revisions

Taxon name	Code	New name	Authority	Max% (coast, inland, ice sheet)	# Occurrences	Planktonic	N_2_-fixing	Prostrate	Solitary	Motile	Mean size (µm)
*Achnanthes carissima*	KARCARIS	*Karayevia carissima*	(Lange-Bertalot) Bukhtiyarova	3, 7, 0	6, 4, 3				1		15.0
*Achnanthes delicatula*	PLNDELIC	*Planothidium delicatulum*	(Kützing) Round and Bukhtiyarova	1, 0, 0	2, 1, 1				1	1	18.5
*Achnanthes lanceolata*	ACHLANCE	*Achnanthes lanceolatum*	Brébisson ex Kützing (1846)	2, 1, 0	6, 4, 3				1	1	20.5
*Achnanthes levanderi*	PSMLEVAN	*Psammothidium levanderi*	(Hustedt) L. Bukhtiyarova and Round	3, 0, 4	10, 2, 9			1	1	1	8.5
*Achnanthes linearis*	ROSLINEA	*Rossithidium linearis*	(W. Smith) Grunow in Cleve and Grunow (1880)	9, 6, 3	11, 7, 7			1	1	1	14.25
*Achnanthes lutheri*	ACHLUTHR	*Platessa lutheri*	(Hustedt) Potapova (2012)	4, 1, 0	4, 4, 0				1	1	11.5
*Achnanthes minutissima	ACDMINUT	*Achnanthidium minutissimum*	(Kütz.) Czarnecki (1994)	6, 7, 7	10, 9, 1			1	1	1	12.5
*Achnanthes oestropii*	ACHOSTRP		(A. Cleve-Euler) Hust.	1, 4, 6	2, 3, 7						12.0
*Achnanthes pusilla	ROSPUSIL	*Rossithidium pusillum*	(Grunow) Round et Bukhtiyarova (1996)	16, 6, 9	13, 8, 10			1	1	1	14.25
*Achnanthes subatomoides	PSMSUBAT	*Psammothidium subatomoides*	(Hustedt) Bukhtiyarova and Round (1996)	11, 4, 7	13, 6, 11			1	1	1	10.5
*Amphora dusenii*	HALDUSEN	*Halamphora dusenii*	(Brun) Leykov	0, 3, 0	1, 5, 1				1		55
*Amphora veneta*	AMPVENET		Kunzing (1844)	0, 7, 1	0, 6, 3				1		32.5
*Anomoneis brachysira	BRABREB	*Brachysira brebissonii*		14, 3, 6	9, 6, 7				1	2	61.5
*Anomoneis brachysira* var *zellensis*	BRAZELL	*Brachysira zellensis*	(Grunow) Round and D. G. Mann	6, 3, 0	1, 1, 0				1		61.5
*Aulacoseira alpigena*	AULALPIG		(Grunow) Krammer	2, 0, 0	5, 0, 0	1					NA
*Caloneis bacillum*	CALBACIL		(Grunow) Cleve (1894)	3, 0, 0	2, 0, 1			1	1	2	31.5
*Cocconeis placentula* var *euglypta*	COCPLAvE		(Ehrenberg) Grunow	4, 0, 0	1, 0, 0			1	1	1	28
*Cocconeis placentula* var *placentula*	COCPLAvP		Ehrenberg (1838)	5, 0, 0	3, 4, 3			1	1		52.75
*Cyclotella* aff *comensis*	CYCafCOM		Grunow in Van Heurck	8, 0, 3	7, 1, 6	1					NR
*Cyclotella bodanica* var *lemanica*	CYCBODvL		(O. Müll. ex Schrot.) H. Bachmann (1903)	0, 0, 2	0, 2, 8	1					NR
*Cyclotella glomerata*	CYCGLOMR		H. Bachmann	1, 0, 0	2, 0, 2	1					NR
*Cyclotella ocellata*	CYCOCELL		Pantocsek (1901)	2, 0, 1	3, 0, 2	1					NR
*Cyclotella pseudostelligera*	CYCPSEUS		Hustedt (1939)	7, 0, 3	6, 2, 6	1					NR
*Cyclotella rossii*	CYCROSSI		Håkansson (1990)	1, 0, 2	3, 0, 3	1					NR
*Cyclotella* spp.	CYCSPEC			2, 0, 0	3, 1, 0	1					NR
*Cymbella descripta	ENPDESCR	*Encyonopsis descripta*	(Hustedt) Krammer (1997)	4, 11, 1	6, 8, 6				1	2	NA
*Cymbella gracilis*	ENMGRACI	*Encyonema gracile*	Ehrenberb (1843)	1, 5, 0	4, 4, 4				1		39.5
*Cymbella microcephala	ENPMICRC	*Encyonopsis microcephala*	Grunow in Van Heurck (1880)	3, 48, 28	10, 11, 11			1	1	2	20
*Cymbella minuta	ENMMINUT	*Encyonema minutum*	(Hilse in Rabenhorst) D. G. Mann (1990)	7, 8, 8	12, 8, 9				1		19.5
*Cymbella silesiaca	ENMSILES	*Encyonema silesiacum*	(Bleisch in Rabenhorst) D. G. Mann (1990)	3, 6, 6	11, 9, 10				1		30.5
*Denticula tenuis*	DENTENUI	*Denticula tenuis*	Kützing (1844)	2, 13, 1	3, 5, 8			1	1	2	18.75
*Diatoma moniliformis*	DIAMONOL		(Kützing) D.M. Williams	0, 1, 0	1, 2, 0	1		1			71
*Diatoma tenuis*	DIATENUI		Agardh (1812)	0, 14, 0	0, 6, 1	1		1			71
*Diploneis elliptica*	DIPELLIP		(Kützing) Cleve (1891)	0, 2, 0	0, 1, 0				1		75
*Diploneis parma*	DIPPARMA		Cleve (1891)	0, 1, 3	1, 2, 3				1		75
*Epithemia sorex	EPISOREX	*Epithemia sorex*	Kützing (1844)	2, 13, 13	5, 8, 11		1	1	1	1	45
*Eunotia exigua*	EUNEXIGU		(Brébisson in Kutzing) Rabenhorst (1864)	3, 1, 0	5, 2, 2				1	1	18
*Eunotia rhomboidea*	EUNRHOMB		Hustedt	2, 0, 0	3, 2, 0				1	1	102.5
*Fragilaria arcus*	HANARCUS	*Hannaea arcus*	(Ehrenb.) Patrick in Patrick and Reimer (1966)	14, 0, 0	2, 0, 0				1		82.5
*Fragilaria brevistriata	PSTBREVI	*Pseudostaurosira brevistriata*	(Grunow) Williams and Round (1987)	15, 14, 9	13, 10, 11			1			20.5
*Fragilaria capucina	FRACAPUC	*Fragilaria capucina*	Desmazière (1825)	6, 6, 5	10, 8, 10						55
*Fragilaria construens*	FRACONST	*Pseudostaurosira construens*	(Marciniak) Williams and Round (1987)	3, 1, 3	10, 4, 8			1			16.5
*Fragilaria construens f *subsalina*	FRACONfS		(Hustedt) E. A. Morales	2, 2, 1	7, 9, 4						20.0
*Fragilaria construens* var *construens*	STRCONfC	*Staurosira construens*	(Ehrenberg) Grunow (1862)	4, 0, 2	3, 0, 1						19.5
*Fragilaria construens var *venter*	STRCONfV	*Staurosira construens* var *venter*	(Ehrenberg) Hamilton (1992)	16, 3, 3	13, 5, 5						19.5
*Fragilaria elliptica*	PSTELLIP	*Pseudostaurosira elliptica*	(Schumann) Edlund, Morales and Spaulding (2006)	7, 8, 5	7, 4, 6			1			16.5
*Fragilaria exigua*	STREXIGU	*Staurosira construens* var *exigua*	(Ralfs) D.M. Williams and Round	10, 8, 2	12, 6, 9						14.0
*Fragilaria nanana* (agg.)	FRANANAN		Lange-Bertalot (1993)	1, 4, 1	4, 2, 4						NA
*Fragilaria parasitica*	PSTPARAS	*Pseudostaurosira parasitica*	(Smith) Morales	2, 0, 0,	4, 0, 0				1		17.5
*Fragilaria pinnata	STLPINAT	*Staurosirella pinnata*	(Ehrenberg) Williams and Round (1987)	19, 10, 16	13, 10, 11						19
*Fragilaria pseudoconstruens*	PSTPSEUC	*Pseudostaurosira pseudoconstruens*	(Marciniak) D. M. Williams and Round (1987)	4, 1, 0	6, 2, 3			1			16.5
*Fragilaria subsalina*	FRFVIRES	*Fragilariaforma virescens*	Grunow	1, 6, 0	3, 2, 0						65
*Fragilaria tenera*	FRATENER		(W. Smith) Lange-Bertalot (1980)	2, 2, 3	32, 2, 3						65
*Frustulia rhomboides var saxonica*	FRURHOvS		(Rabenhorst) De Toni	0, 3, 0	0, 1, 0				1	2	100
*Gomphonema accuminatum*	GOMACCUM		Ehrenberg (1832)	2, 1, 1	6, 4, 5			1	1	2	70
*Gomphonema clavatum*	GOMCLAVA		Ehrenberg (1832)	4, 0, 0	1, 0, 0				1		57.5
*Meridion circulare*	MERCIRCR		(Greville) C. Agardh(1831)	4, 0, 0	4, 0, 0						46
*Navicula cocconeiformis	CAVCOCON	*Cavinula cocconeiformis*	(Gregory ex Greville) Mann and Stickle in Round, Crawford and Mann (1990)	4, 4, 4	12, 5, 7				1	1	26
*Navicula cryptocephala*	NAVCRYPC		Kützing (1844)	4, 4, 5	9, 9, 11				1	2	26.5
*Navicula cryptonella	NAVCRYPT		Lange-Bertalot (1985)	2, 5, 4	4, 10, 9				1		27
*Navicula pseudoscutiformis	CAVPSEUS	*Cavinula pseudoscutiformis*	(Hustedt) Mann and Stickle in Round, Crawford and Mann (1990)	2, 11, 11	7, 6, 11				1	1	25.75
*Navicula radiosa	NAVRADIO		Kützing (1844)	2, 1, 1	7, 7, 7				1	2	56
*Navicula schmassmannii*	NAVSCHMS		Hustedt (1943)	7, 0, 0	9, 0, 1				1	1	8.0
*Navicula subtilis*	NAVSUBTIL		Brébisson ex Kützing	2, 11, 8	3, 6, 3				1		50
*Nitzschia amphibia*	NITAMPHB		Grunow (1862)	0, 2, 1	0, 3, 3			1	1	2	31
*Nitzschia cf alpina*	NITcfALP		Hustedt (1943)	0, 0, 6	1, 2, 1				1	2	30.0
*Nitzschia fonticola	NITFONTC		(Grunow in Cleve and Grunow) Grunow in Van Heurck (1881)	16, 45, 26	11, 10, 11			1	1	1	37.5
*Nitzschia frustulum*	NITFRUST		(Kützing) Grunow in Cleve and Grunow (1880)	13, 12, 8	8, 9, 10			1	1	2	32.5
*Nitzschia gracilis*	NITGRACI		Hantzsch (1860)	1, 2, 1	3, 4, 8				1		70
*Nitzschia inconspicua	NITINCON		Grunow (1862)	12, 6,20	9, 5, 10				1		12.5
*Nitzschia palea*	NITPALEA		(Kützing) W. Smith (1856)	11, 11, 11	13, 10, 11			1	1	2	42.5
*Nitzschia paleacea*	NITPALCE		(Grunow in Cleve and Grunow) Grunow in Van Heurck (1881)	1, 3, 0	1, 3, 1				1	2	31.5
*Nitzschia valdestriata*	NITVALDS		Aleem and Hustedt (1951)	1, 4, 0	1, 3, 0				1		9
*Tabellaria fenestrate*	TABFENES		(Lyngb.) Kütz (1844)	2, 0, 0	6, 0, 0	1					57.5
*Tabellaria flocculosa	TABFLOCC		(Roth) Kützing (1844)	39, 3, 25	13, 5, 11	1		1			68

The maximum relative abundance in any one sample is presented (Max %), the number of samples that the taxon is present in (# occurrences) and taxa are categorised by functional attributes (1 = yes for planktonic, N_2_-fixing, prostrate, solitary; 1 = slightly motile; 2 = moderately motile), mean sizes are presented in the final column. The size and attribute data is collated from (Kelly et al. 2005; Spaulding et al. 2010) except where not available (NA) or not required (NR; planktonic taxa). * Taxa which are present at ≥5% and present in ≥20 of the samples, used in the epilithon sums for Fig. 8

Lake water was collected for chemical analysis concurrent with the diatom samples and, in addition, a further sampling for water chemistry was conducted from under the lake ice in April 2011 (Whiteford et al. 2016). The mean of these three samples was calculated for each lake to provide an overview of regional differences in water chemistry. This water chemistry dataset has been previously published in Whiteford et al. (2016), but here we include only values from the lakes sampled for epilithon, which is a subsample of the broader survey. Water chemistry sampling and analysis followed standard procedures as detailed in Hogan et al. (2014).

### Numerical analyses

Patterns in water chemistry across the sampling transect were explored using principal components analysis (PCA) of the mean water chemistry data. Data were first checked for homogeneity of variance and transformed if necessary (indicated in Table 1). PCA and other ordinations were performed on CANOCO version 5 (Ter Braak and Smilauer 2012). Diatom assemblages along the transect were explored for each sampling occasion (Spring 2011, Summer 2010) using detrended correspondence analysis (DCA), after establishing that axis 1 gradient lengths exceeded 2.5 SD and thus unimodal analysis was appropriate (Leps and Smilauer 2003). Canonical correspondence analysis (CCA) was used to analyse relationships between diatom taxa and water chemistry variables; in this analysis we paired diatoms with water chemistry values sampled on the same occasion, performing independent analyses for summer 2010 and Spring 2011. Because the sampling was focused in three distinctive regions, we included a measure of spatial autocorrelation in a partial CCA to evaluate the influence of spatial structure on the overall analysis (Borcard et al. 1992). A Principal Coordinates of Neighbour Matrices (PCNM) analysis of the UTM site coordinates was used to summarise spatial patterns in the data (Dray et al. 2006). Because almost all lakes in the dataset are hydrologically disconnected from one another, a more sophisticated network analysis was unnecessary (Blanchet et al. 2008). The variance partitioning with PCNM feature of CANOCO version 5 was used with manual forward selection (1000 Monte Carlo permutations) being used to establish which principal coordinates were significantly correlated with the diatom data (in this case the second principal coordinates axis PCO2). PCO2 was then used as a covariable in a partial CCA with manual forward selection to eliminate the chemical variables that were not significantly correlated with the diatoms (*p* < 0.05). Variables with the highest variance inflation factors (VIF) were sequentially removed from the partial ordination until all VIFs were <21 (Hall et al. 1999). The final analyses with the remaining environmental variables were displayed as a CCA to understand patterns among species and environmental factors, and then as a partial CCA with variance partitioning analysis (VPA) to determine the proportion of variance explained by environmental, spatial or both (environmental + spatial) components.

Diatom assemblage structure was explored by calculating richness (total number of taxa per sample) and diversity using the Shannon-Weiner diversity index where zero denotes minimum diversity. The ecological characteristics of all diatoms at ≥2% abundance were quantified with reference to Kelly et al. (2005) and Spaulding et al. (2010). Diatoms were characterised as follows based on growth habits; (1) planktonic (acknowledging that presence in the epilithon is probably caused by settling of planktonic taxa, but that some taxa can live in both benthic and planktonic habitats), (2) potential ablility to fix atmospheric nitrogen through symbiotic cyanobacterial associations (DeYoe et al. 1992), (3) prostrate (grows lying flat on the rock surface), (4) solitary (usually grows alone, but recognizing that some diatoms are able to grow both alone or in colonial form), and (5) moderately motile (not including diatoms that are classified as slightly motile). Diatom sizes were also estimated by calculating the mean of the size ranges given in Kelly et al. (2005), or other sources such as Krammer and Lange-Bertalot (1986–1991) when necessary. This information was summarized by presenting the % of each ecological category and, for diatom size, a weighted mean of the frustule sizes in each diatom sample. When ecological information was unavailable for taxa (see Table 2), they were excluded from the integrated sums.

### Epilithic diatoms in lake sediments

In three lakes (one from each region) for which HON-kajak sediment cores had been sampled from the deepest part of the lake basins, we compared the relative abundance of diatoms in the uppermost 0.5 cm of sediment with the epilithic samples from the same lake to determine potential representation of epilithon in lake sediments. The lakes selected for this analysis were, as far as possible, similar in size and depth and included AT6 (coast), SS1381 (inland) and SS904 (ice sheet margin). For this analysis due to taxonomic difficulties *Cyclotella stelligera*, *Cyclotella pseudostelligera* and *Cyclotella stelligeroides* were grouped as ‘*Cyclotella stelligera* complex’. We then used data from previously published Holocene diatom sequences in this region to test the application of the epilithic diatoms as palaeoenvironmental indicators (Law et al. 2015). Detailed site descriptions, information on the coring and construction of the sediment chronologies are given in Anderson et al. (2012) whereas information on the diatom preparation techniques are given in Law et al. (2015). To assist in the interpretation of the Lake SS8 record, sedimentary concentrations of myxoxanthophyll, a carotenoid from cyanobacteria are also presented. Pigment analysis methods followed those in McGowan et al. (2012) as outlined in Liversidge (2012).

## Results

**Fig. 2:**
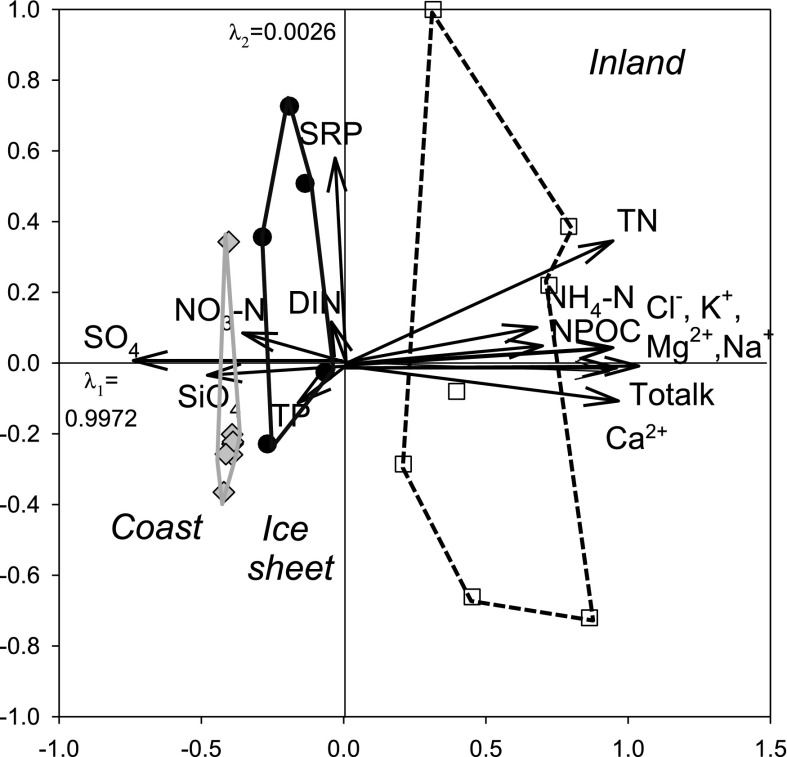
PCA of mean water chemistry of study lakes. Mean values were calculated from sampling during July–August 2010, April 2011 and May–June 2011. Samples from the coast are shown in *grey diamonds*, from the ice sheet in *black circles* and from inland area in *white squares*, with the lines encapsulating the samples

There was a pronounced difference in chemical composition of lake groups from each area (Table 1; Fig. 2). Coastal lakes had lower major ion concentrations (K^+^, Na^+^, Mg^2+^, Ca^2+^, Cl^−^) and lower total alkalinity, but higher concentrations of nitrate-nitrogen, silicate and sulphate ions. Inland lakes had higher concentrations of major ions, total nitrogen, ammonium and non-particulate organic carbon (NPOC) and were more diverse in terms of chemical composition than lakes from other areas. Lakes close to the ice sheet were chemically intermediate, but had more in common with the coastal than the inland sites.

**Fig. 3:**
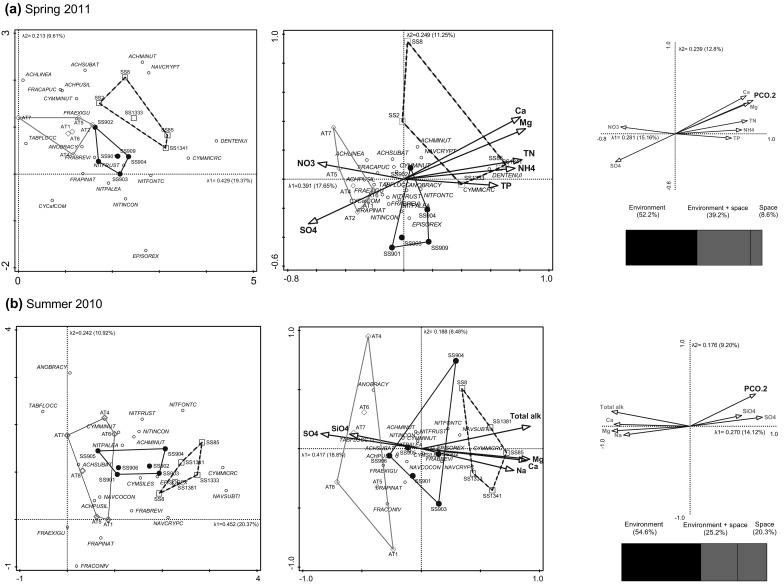
DCA (*left panel*) of epilithic diatom assemblages (grouped by lake region), CCA (*middle panel*) showing relationships with limnological parameters and partial CCA (*right panel*) of the same environmental variables with space (PCO2) as a covariable, which was used to partition variance among environment, environment + space and space categories (shown in the *bar chart*) as sampled in **a** Spring 2011 and **b** Summer 2010. The diagram display is restricted to the 20 diatom taxa with the largest weight on the analysis as determined in CANOCO 5

Of the 185 epilithic diatom taxa from 27 genera that were identified, 75 had a relative abundance of >2% (Table 2). In Spring 2011 the most abundant diatoms in the coastal lakes were *Pseudostaurosira brevistriata*, *Rossithidium pusillum*, *Staurosirella pinnata* and *Tabellaria flocculosa* (Table 2; Fig. 3a). *Encyonopsis microcephala* dominated the inland lakes with *Diatoma tenuis*, *Denticula tenuis* and *Nitzschia fonticola* also common. At the ice sheet, *E. microcephala* and *S. pinnata* were co-dominant with *Epithemia sorex*, *N. fonticola*, *Nitzschia inconspicua*, *Nitzschia palea* and *T. flocculosa*, being common. The most abundant diatom in lakes at the coast during Summer 2010 was *T. flocculosa* with *R. pusillum*, *Staurosira construens* var. *venter*, *S. pinnata* and *N. fonticola* also being abundant (Table 2; Fig. 3b). At inland sites *E. microcephala* was dominant, with *Encyonopsis descripta*, *N. fonticola*, *Navicula subtilis* and *P. brevistriata*, being abundant in some sites. Summer samples from ice sheet margin lakes were dominated by *E. microcephala* with *E. sorex*, *N. fonticola*, *S. pinnata* and *T. flocculosa* being locally abundant. Across all sites the most widespread and abundant diatoms were *E. microcephala*, *N. fonticola* and *S. pinnata* (Table 2).

Diatom assemblages were most closely and significantly correlated with nutrient variables in the spring (Fig. 3a). Elevated concentrations of total nitrogen, total phosphorus, ammonium and major ions (Ca^2+^, Mg^2+^) were positively correlated with axis 1 (λ = 0.391) and associated with inland lake diatom assemblages (*Denticula tenuis*, *E. microcephala*). Coastal lakes were negatively correlated with axis 1, had elevated concentrations of nitrate and sulphate ions and assemblages where *P. brevistriata*, *Rossithidium linearis*, *R. pusillum* and *T. flocculosa* were common. Ice sheet lakes were intermediate between the two other areas, but had relatively higher proportions of *E. sorex* and *N. inconspicua*. CCA showed that major ions were the most significant correlates with summer epilithon (Fig. 3b). Alkalinity and major ions (Ca^2+^, Mg^2+^, Na^+^) were positively correlated with axis 1 (λ = 0.4171) and with the inland lake diatoms, predominantly *E. microcephala.* Silicate and sulphate ions were more abundant in the coastal lakes, negatively correlated with axis 1 and associated with a greater variety of diatoms (*Brachysira brebissonii*, *R. pusillum*, *Staurosira construens* var *exigua*, *S. pinnata* and *T. flocculosa*). Ice sheet lakes were intermediate along this axis 1 gradient. Water chemistry variables were highly spatially organised (Fig. 3), but variance partitioning analysis (VPA) using the partial CCA indicated that environmental variables uniquely accounted for 52.2 and 54.6% of the variance in spring and summer respectively, whilst space + environment accounted for 39.2 and 25.2% of variance. Spatial factors uniquely accounted for 8.6 and 20.3% of variance in the spring and summer periods. Thus, environment and (environment + space) accounted for more variance overall during the spring than the summer.

**Fig. 4:**
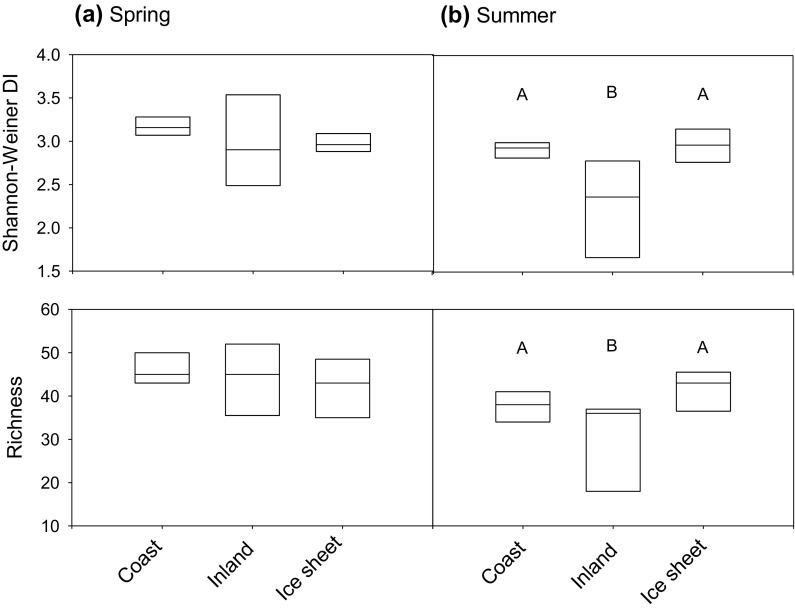
*Box plots* showing diversity as measured by the Shannon-Weiner Diversity Index (*top panel*) and richness (*lower panel*) of epilithic diatom taxa sampled during **a** Spring 2011 and **b** Summer 2010. Significant differences (*p* < 0.05) between lake regions from ANOVA and Tukey’s Least Significant Difference tests are indicated by *different letters* (*A* versus *B*)

Epilithic diatom assemblages were generally more diverse and had greater numbers of taxa during the spring than the summer (Fig. 4a). Diatom assemblage richness and diversity was higher in the coastal lakes during Spring 2011, but not significantly so. During Summer 2010, diatom assemblage richness and diversity was significantly lower in the inland lakes relative to coastal and ice sheet lakes (Fig. 4b). Inland lakes also showed a more pronounced decline in richness and diversity in the summer relative to the spring, whereas the decline in the coastal lakes was more subtle and it increased slightly in the ice sheet lakes.

**Fig. 5:**
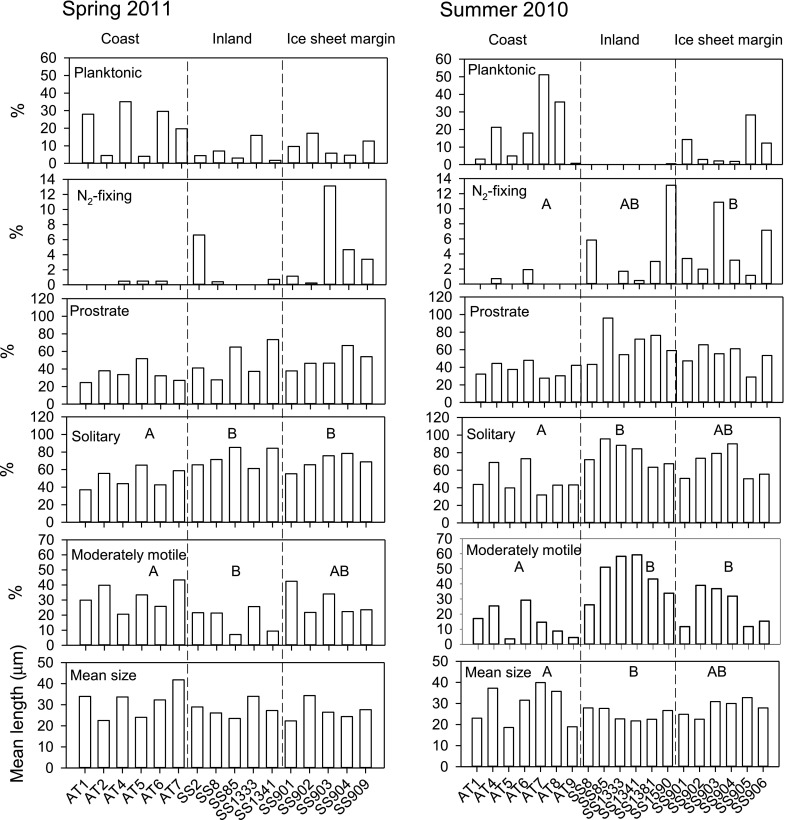
Relative abundance of diatom growth forms in the study lakes in Spring 2011 and Summer 2010. Lakes are ordered by study area across the region of southwest Greenland (coast-inland-ice sheet margin) with each area separated by *dashed horizontal lines*. Significant differences (*p* < 0.05) between lake regions from ANOVA and Tukey’s Least Significant Difference tests are indicated by *different letters* (*A* versus *B*)

When categorised by functional attributes, assemblage characteristics were more distinctive in each lake region during the summer (more significant differences among lake regions) than the spring (Fig. 5). Proportionally more planktonic diatoms were found on the rock surfaces of coastal lakes. During the summer, planktonic diatoms were absent from rock scrapes of almost all inland sites. The distribution of N_2_-fixing *Epithemia sorex* was distinctive, with low relative abundance or absence in the coastal lakes, and significantly higher proportions in most ice sheet margin lakes and some inland sites, particularly during the summer. There was a less distinctive pattern of prostrate diatoms, but generally lower contributions of this diatom type in the coastal lakes. Solitary diatoms were significantly less abundant in coastal lakes than other areas. The pattern of motile diatoms changed seasonally; inland sites having the lowest proportions of motile diatoms in the spring, but highest during the summer. Mean diatom frustule length in the coastal lakes was generally greater than in the other areas with the differences becoming more pronounced during the summer.

**Fig. 6:**
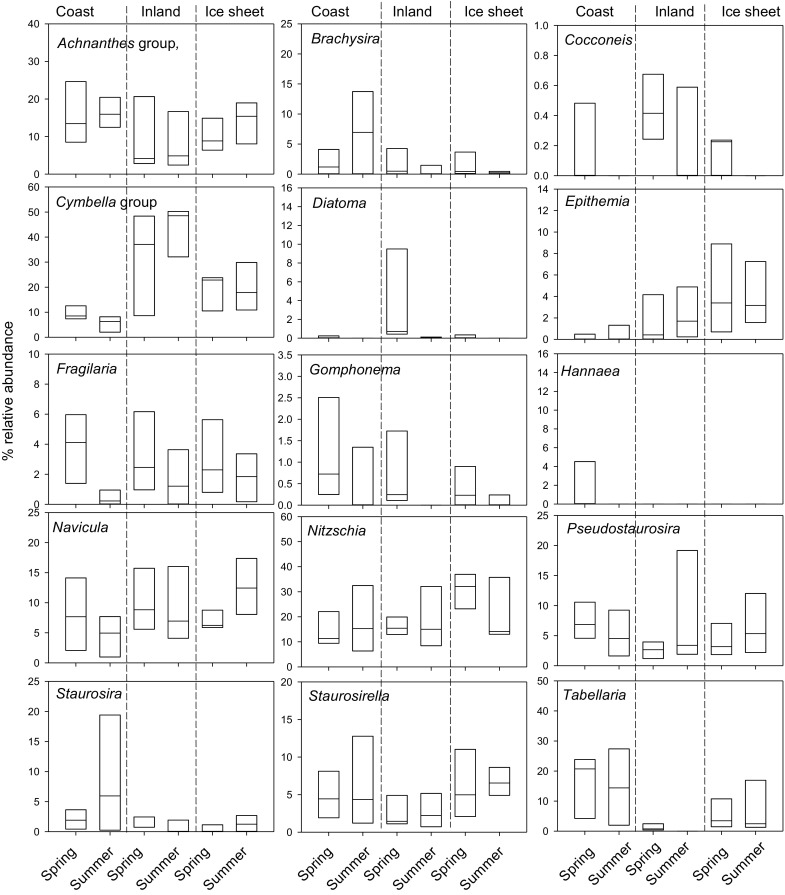
Epilithic diatom variability among study areas (coast-inland-ice sheet margin) and sampling occasion (spring, summer). Diatoms are summed by genera or families including the *Achnanthes* group (*Achnanthes*, *Achnanthidium*, *Platessa*, *Karayevi*a, *Psammothidium*, *Rossitihidium*, *Planothidium* spp.) and *Cymbella* group (*Encyonema*, *Encyonopis*)

Comparisons of aggregated diatom genera across seasons and regions showed a variety of patterns (Fig. 6). There were lower relative abundances of *Cocconeis*, *Diatoma*, *Fragilaria* and *Gomphonema* spp. in summer relative to the spring, but all of these genera were present in low relative abundances in the assemblage (<10%). Other seasonal responses differed among study areas; for example *Brachysira* spp. increased between spring and summer in coastal sites, but declined in the inland and ice sheet sites and the converse was true for *Pseudostaurosira* spp. Certain genera changed most clearly along the spatial transect with indistinct seasonal trends. *Epithemia*, *Navicula* and *Nitzschia* spp. increased in abundance from the coast- inland- ice sheet margin, whereas the *Achnanthes* group, *Hannaea*, *Staurosira* and *Tabellaria* spp. were highest in the coastal sites, and inland the *Cymbella* group were significantly higher and *Staurosirella* were lower.

### Epilithon- surface sediment comparisons

**Fig. 7:**
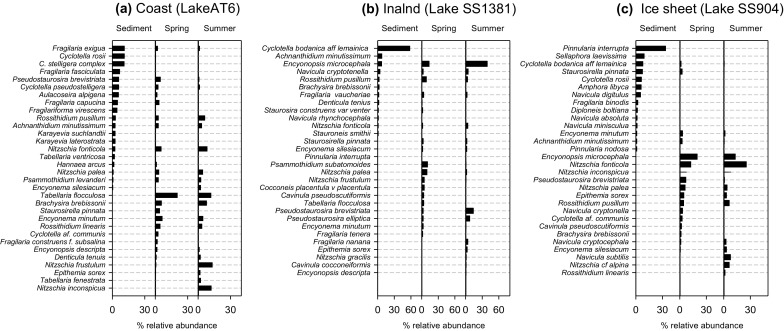
Comparisons of diatom assemblages from profundal surface sediments and epilithon scrapes of study lakes **a** AT6 (coast) **b** SS1381 (inland) lakes and **c** SS904 (ice sheet margin). Sediment cores were extracted from the deepest part of each lake for comparison to epilithon assemblages sampled during Spring 2011 and Summer 2010. The most abundant diatoms from each respective sample are selected

Diatoms detected in the epilithon samples comprised between 15 and 40% of the taxa within the deep-water sedimentary diatom assemblage of the three lakes. While many of the common taxa were preserved in the surface sediments, several were absent (Fig. 7). In Lake AT6 of the 67 diatom taxa in the sediments, 27 were potentially from the epilithon, with a further three being found on rocks, but likely originating from the plankton (*Aulacoseira alpigena*, *C. pseudostelligera* and *C. rossii*/*ocellata* complex) (Fig. 7a). This amounted to 41% of the relative abundance of diatoms in sediments from lake AT6 being epilithic taxa (excluding the probable planktonic ones). The main “non-epilithic” diatoms (as defined by our rock scrapes) in sediments of this lake included *C. stelligera* complex, *Fragilaria fasiculata*, *Fragilariaforma virescens*, *Karayevia suchlandtii* and *Karayevia laterostrata*. Common (>3%) epilithic taxa not/under- represented in the sediment were *T. flocculosa* (12–20% in rock assemblages), *N. frustulum* (1–13%), *N. inconspicua* (0–12%), *B. brebissonii* (6–8%), *E. minutum* (5–7%) and *R. linearis* (4–5%). In the inland lake SS1381, 12 out of 29 taxa were epilithic, representing a relative abundance of 25% (Fig. 7b). In this lake, the majority of the sedimentary diatoms were planktonic taxa with *C. bodanica* var. *lemanica* comprising 60% of the assemblage and *Achnanthidium minutissimum* also being abundant. The most abundant epilithic species not recorded in sediments included *P brevistriata* (3–39% of the rock assemblage), *Psammothidium subatomoides* (1–10%), *P. elliptica* (3–8%), *Nitzschia palea* (2–10%), *N. frustulum* (1–5%), *Cocconeis placentua* var. *placentula* (0.5–5%), *Fragilaria nanana* (0–4%), *Cavinula pseudoscutiformis* (0–4%) and *T. flocculosa* (0–4%). Out of 33 taxa in ice sheet lake SS904 sediments, five were recorded on rocks with a further one (*C. bodanica* var. *lemanica*) probably deriving originally from the plankton (Fig. 7c), making up a relative abundance of 10% of the assemblage (excluding possible plankton-derived taxa). Abundant diatoms in the sediments that were not detected in the epilithon scrapes were *Pinnularia interrupta*, *Sellaphora laevissima*, *S. pinnata* (although present in the epilithon of other lakes; Table 2), *Amphora libyca*, *Navicula digitulus* and the planktonic *C. bodanica* var. *lemanica* and *C. rossii*. Common epilithon diatoms not detected in the sediments included a range of *Nitzschia* species (*N. fonticola* (13–26% of rock assemblages), *N. inconspicua* (7–8%), *N. frustulia* (4–7%) *N.* cf. *alpina* (0–6%), *N. palea* (4–6%)), *E. microcephala* (13–20%), *P. brevistriata* (1–7%), *Navicula subtilis* (0–8%) and *R. pusillum* (4–6%).

## Discussion

The pronounced gradients in water chemistry identified in this study have been demonstrated in previous surveys around Kangerlussuaq, showing that lakes located inland are more enriched in major ions, nutrients and non-particulate organic carbon (NPOC) (Anderson et al. 2001; Anderson and Stedmon 2007). These patterns are largely driven by local climate where low precipitation: evaporation ratios in the inland and ice sheet marginal areas, together with aerial deposition of dust from the glacial sandur plains have led to the concentration of salts and dissolved organic carbon (DOC), low hydrological flushing rates and strong recycling of nutrients (total N and P) (McGowan et al. 2003; Anderson et al. 2008). This dependence on in-lake nutrient recycling leads to the divergence in chemical characteristics across the region and so inland lakes are more chemically diverse than lakes in other areas. In contrast the coastal area has ca. three-times greater winter snowpack depth leading to increased snowmelt and, because of active hydrological inputs, coastal lakes have much higher flushing rates (Curtis et al., unpublished). The consequence is more dilute and slightly acidic waters, but higher concentrations of reactive N (as nitrate) and sulphate, which is predominantly delivered in the spring from atmospheric deposits accumulated in over-winter snow pack (Hogan et al. 2014; Whiteford et al. 2016). Most nitrogen in inland lakes is present as ammonium and total N (including organically-bound) forms, consistent with a strong reliance on in-lake recycling and retention of nitrogen with the oxygen-depleted bottom waters in many of the inland lakes where ammonium is replete, deriving from the decomposition of organic matter. Higher silicate concentrations in coastal lakes are linked with the more intense weathering of the gneiss bedrock in the region and efficient delivery to lakes due to the wetter conditions (Anderson et al. 2012). The ice sheet margin lakes are intermediate in terms of water chemistry; there is limited precipitation but cooler conditions which slow down evaporation rates limiting the evapo-concentration of major ions and DOC (Anderson and Stedmon 2007) but greater inputs of phosphorus from aeolian dust.

Previous training set studies of surface sediment diatom and chironomid assemblages show that chemical differences within this lake district correlate with changes in biota, although they included the chemically-distinct “saline” lakes which were excluded from this study (Brodersen and Anderson 2002; Ryves et al. 2002). See Pla and Anderson (2005) for comparisons of saline and non-saline chrysophyte cyst assemblages. Here, differences among littoral epilithic diatom assemblages in the three areas (coast, inland, ice sheet margin) confirmed that they were responding to the different lake conditions, even along a narrower freshwater chemistry gradient; variance from CCA axes 1 and 2 explained about 25% in Ryves et al. (2002) compared with values in this study of 29% in the spring and 27% in the summer. The pCCA (Fig. 3) analysis identified that epilithic diatoms were most closely correlated with nutrients in the spring and with major ions in the summer (when nutrient concentrations were lower), because nutrients are rapidly depleted from these lakes after ice-off (Whiteford et al. 2016). Such observations are commensurate with the idea that nutrients in snowmelt and meltwater delivery is an important determinant of epilithic diatom assemblages (Catalan et al. 2002). Nevertheless, silicate was positively correlated with diatoms at the coast during the summer, suggesting that the greater availability of this nutrient in these more rapidly flushed lakes may be important in structuring the diatom assemblages, resulting in greater abundance of heavily silicified diatoms (*Tabellaria*, *Staurosira* and *Staurosirella*). The partial CCA and VPA demonstrated that more than half of the variance explained by the CCA could be attributed solely to environmental factors, confirming that diatoms are robust indicators of water chemistry variables. Additionally however, just under half of the variance was correlated with spatial or (environmental + spatial) factors, (i.e. chemical and other factors co-varied with lake region). Thus, the relationships between environmental variables and diatoms that we identified might be partly be explained by un/measured variables which also co-varied with location (for example fish abundance which is lower in the hydologically-isolated inland/ice sheet lakes) (Juggins 2013). The analysis further highlights how sampling during different seasons can influence correlations, with implications for palaeoecological interpretations.

### Functional attributes of epilithic diatoms

“Pioneer” diatom taxa which are present during the early spring period could provide information about phenology, and therefore have potential as palaeoclimate indicators. Unfortunately only a few diatoms showed consistently higher abundances in the spring (*Gomphonema*, *Cocconeis*, *Diatoma* and *Fragilaria* species), and they comprised low proportions of the total assemblages. The presence of *Gomphonema* is consistent with observations that it is a successful colonising species in resource replete environments (McCormick and Stevenson 1991), growing shortly after snowmelt when reactive nitrogen is abundant (Whiteford et al. 2016). *Hannaea arcus* is strongly associated with the spring period, but only in some of the coastal lakes. This diatom has previously been used as an indicator of riverine inputs (Douglas et al. 1996), demonstrating that surface inflows into lakes at the coast are most active after the spring snowmelt period. Overall though, spring assemblages included a diverse mix of adnate (*Achnanthes*, *Cocconeis*), motile (*Nitzschia*, *Navicula*, *Brachysira*, *Cymbella*), stalked (*Gomphonema*) and unattached/loosely attached colonial taxa (*Tabellaria*, *Pseudostaurosira*, *Staurosira*, *Staurosirella*) alongside planktonic taxa (*Diatoma*, *Cyclotella*) which had settled onto rocks from the lake seston. The spring sampling took place within a few weeks (inland sites), or days following ice-off (coastal, ice sheet margin lakes) and suggests that biofilm and diatom assemblage succession to a mature three-dimensional community takes place over short timescales of days-weeks (Peterson 1996) and may contain ‘memory’ of overwintering taxa from the previous growth season (Quesada et al. 2008). Identification of early colonizers in these lakes might have been better detected by sampling lakes prior to ice-off when only moating had occurred. This only happened at some of the sites in the coastal area where remnants of ice remained, and is practically difficult to achieve when access to lakes is restricted and ice-off dates are difficult to predict.

Comparison of the spring and summer epilithon samples suggests that successional trajectories differed in each lake region, and that seasonal differences were most pronounced in the inland sites. Species diversity and richness declined and motile species (mostly *Encyonopsis microcephala*, *Nitzschia* and *Navicula* species) became more dominant during the summer in the inland and ice sheet lakes. *E. microcephala* is a common component in biofilms in the later stages of diatom succession (Stevenson et al. 1991; Barbiero 2000). We infer that low silicate concentrations during the summer and the availability of nitrogen and phosphorus in organically bound forms at the inland sites led to the development of more complex biofilms with proportionally more non-siliceous algae, as confirmed by previous periphyton experiments (Hogan et al. 2014). The greater availability of dissolved organic matter (NPOC) in the inland sites implies more intense heterotrophic microbial processing. Complex biofilms are well suited to motile diatom taxa because they can move up and down for light harvesting, and some *Nitzschia* species can secrete extracellular enzymes to decompose organic compounds for heterotrophic nutrition (Tanaka and Ohwada 1988; Tuchman et al. 2006). It is likely that they compete very effectively in highly organic biofilms and in lakes with abundant organic C because exopolymers are also important for diatom motility (Smith and Underwood 1998). In line with this observation, contamination of an Arctic lake with organic sewage was shown to increase the relative abundance of *Navicula* and *Nitzschia* taxa (Michelutti et al. 2007b). It is noteworthy that the replacement of *Fragilaria*/fragilarioid taxa with *Nitzschia* has been observed in several Arctic lakes in recent decades (Antoniades et al. 2005b; Keatley et al. 2006), raising the question that such shifts may indicate organic enrichment of lakes, which could be associated with important Arctic processes such as permafrost melt (Pokrovsky et al. 2011) and ‘Arctic greening’ (McGowan et al. 2016).

As well as more motile species, more prostrate diatom taxa were also observed in the inland lakes later in the growth season. Prostrate forms are suited to growth as epiphytes on algal filaments and so may be an adaptation to growth in complex microbial mats (McCormick and Stevenson 1991). Grazing pressure might also be a structuring force for diatoms in the inland lakes because most lack fish due to hydrological isolation. The absence of top predators, longer and warmer ice-free seasons and the alkaline conditions which are more suitable for calcareous organisms such as molluscs should make these lakes especially suited to rich and diverse invertebrate grazer communities (Bennike 2000; Brodersen and Anderson 2002; Reuss et al. 2014). Thus, adnate growth forms may be an adaptation for grazer avoidance (Jones et al. 2000). The other very marked seasonal pattern in the inland lakes is the absence of planktonic diatoms in the summer period, associated with epilimnetic depletion of silicate (and other nutrients). For example, *Diatoma tenuis* is common in the plankton of meromictic saline lakes in this region (Willemse et al. 2004) and thus is categorized as a halophilic diatom in the training set of Ryves et al. (2002). Its presence in these lakes (conductivity < 605 µS cm^−1^) demonstrates that it is most likely not driven by salinity, but rather an ability to grow rapidly after ice out when nutrients are available until silica is depleted (Morabito et al. 2003). Overall, the hydrological isolation and warm summer conditions of the inland lakes appear to have multiple influences on epilithic diatoms by limiting external nutrient delivery (especially silicate), increasing the dependence on in-lake cycling of organically-bound nutrients and modification of food web structure through the absence of top predators.

Some diatoms were apparently influenced more by the overall conditions within lake regions than by seasonal changes. For example, nitrogen-fixing diatoms, represented solely by *E. sorex* in this dataset, were most abundant in ice sheet lakes and common in the inland sites. *E. sorex* is known to host endosymbiotic N_2_-fixing cyanobacteria (DeYoe et al. 1992) and its presence is consistent with our observations that delivery of reactive nitrogen in this region is two times lower than at the coast (Curtis et al. unpublished). Bioassay experiments confirm that periphyton growth in the summer is nitrogen-limited in the ice sheet lakes and limited by nitrogen and phosphorus inland, explained by the enhanced supply of P to ice sheet lakes in dust which blows from fluvioglacial deposits from the ice sheet (Hogan et al. 2014). Highly silicified *Tabellaria*, *Staurosira* and *Staurosirella* were abundant in both seasons in the coastal lakes. *Tabellaria flocculosa* is often associated with dilute and acidic waters, and “acid pulses” in spring snowmelt in upland sites (Knudson and Kipling 1957; Cameron et al. 1999; Štefková 2006). However, our analyses indicate that perennial rivers flowing into the coastal sites (several served by snow banks higher in the catchment) throughout the summer likely maintained optimal conditions for growth of these taxa. The close correlation with sulphate ions in the CCA suggests that *T. flocculosa* is strongly responsive to water chemistry and it is common in the epilithon of acidified and subarctic lakes (Albert et al. 2009).

### Representation of littoral epilithic diatoms in sediments

Sampling of individual habitats can provide information about habitat preferences, but many benthic diatoms grow on multiple substrate types (Lim et al. 2001b). Therefore, although this study has identified the diatoms which may grow on rock surfaces at the lake margins, it has not demonstrated habitat specificity because other benthic habitats (including deeper epilithon) were not sampled (Cantonati et al. 2009). The discussion below identifies such diatoms as shallow water epilithon, but acknowledges the caveat that other habitats were not sampled. Epilithic diatoms made up a rather small proportion of the lake sedimentary diatom assemblages (between 10 and 41%). Lake bathymetry is an important influence on the proportions of epilithic diatoms in sediments because the subfossil diatoms derive from plankton and a variety of benthic habitats (Stone and Fritz 2006; Anderson and Battarbee 1994). Photic zone depth is similar across this lake district (mean and standard deviation of 17 ± 0.5 m at the coast, 15.2 ± 0.8 m inland and 18.6 ± 0.9 m at the ice sheet) (Whiteford et al. 2016) and extends to the bottom of most lakes (SS904 maximum depth 11.4 m; SS1381, 18.5 m; AT6 23 m) indicating that most of the benthic region in each lake received sufficient light during the ice-free season to support photosynthetic communities. Thus non-planktonic diatoms usually comprise around 50% of the total sedimentary diatom assemblage in many lakes in this region (Ryves et al. 2002) and benthic production is important in these lakes. The very low representation of epilithic diatoms in the ice sheet lake SS904 (10%) may be explained by the greater proportion of other benthic algae including *Pinnularia interrupta* and *Sellaphora leavissima.* Many *Pinnularia* species are epiphytic on mosses in arctic lakes (Michelutti et al. 2006) and *S. leavissima* is known to be epipelic, suggesting that SS904 may have well developed aquatic plant coverage.

Taphonomy of sedimentary diatom assemblages might be influenced by the dissolution of frustules and those from nearshore environments are particularly prone to resuspension from turbulence and breakage, making them susceptible to dissolution (Ryves et al. 2006). In contrast, planktonic diatoms may be quite rapidly deposited into sediments, sometimes assisted by sinking in zooplankton faecal pellets (Cameron 1995). Such differences in transport efficiency would make littoral epilithic diatoms more susceptible to modification by the chemical conditions in lakes, and the lower silicate concentrations and higher salt concentrations/alkalinities in the inland and ice sheet lakes may have increased the dissolution of frustules (Ryves et al. 2006). The low sedimentation rates, typical of Arctic lakes, also increases exposure to unfavourable chemical conditions, and thus periods of poor diatom preservation are common in sediment cores from the inland lakes (Law et al. 2015). The sediment-epilithon comparisons suggest that there is selective loss of certain taxa from the epilithic communities. In particular, *Tabellaria flocculosa*, common in the coastal lakes’ epilithon, is under-represented in the sediments in Lake AT6, and it is possible that this colonial and loosely attached/unattached diatom is susceptible to washout in these lakes which are rapidly flushed. *Nitzschia* species are particularly under-represented in sediments of the inland/ice sheet lakes. *Nitzschia* are renowned for their low representation in many lake sediments, possibly associated with their lightly silicified frustules which dissolve easily (Ryves et al. 2003; Battarbee et al. 2005). The other taxon that is important in epilithon and yet poorly represented in sediments in the ice sheet lakes is *Encyonopsis microcephala.* It appears therefore, that solitary diatoms (*Encyonopsis*, *Nitzschia*) are especially susceptible to taphonomic processes, and their smaller surface area relative to colonial growth forms might expose them to chemical dissolution and breakage. In lakes such as these with slow sedimentation rates, the diatoms deposited in surface sediments are mixed together with existing sedimentary assemblages and so the cumulative upper sediments can differ substantially from the source assemblage (Cameron 1995). This effect is particularly pronounced if contemporary algal deposits are different from those previously deposited as may be the case in this region of Greenland which is now rapidly changing (Anderson et al. 2016).

The surface sediment comparisons of the three lakes across the lake district indicate greater abundance of epilithic diatoms in the coastal lake, and of epilithic + epipelic diatoms in the ice sheet lake, each of which lose ice at a slower rate than the inland lakes. This agrees with suggestions that lakes which undergo prolonged periods of moating should have a greater proportion of periphyton relative to plankton (Douglas and Smol 1999; Catalan et al. 2002). When lakes are moating, epilithic algae in littoral areas have access to nutrients from snowmelt, which might provide a competitive advantage over planktonic diatoms which would be comparatively constrained by light availability under ice and lack of wind mixing (Bertilsson et al. 2013). Furthermore, snowmelt is three times greater at the coast leading to a two-fold greater flux of inorganic nitrogen into the coastal lakes than the inland area (C. Curtis, unpublished), which could account for the greater proportions of littoral epilithic diatoms (Whiteford et al. 2016). The proportional contribution of littoral epilithon to overall diatom assemblages is lowest at the ice sheet lake, where epipelic diatoms are more common. We propose that the greater numbers of plant- and sediment-associated diatoms in the ice-sheet lake are instead utilizing sedimentary nutrient sources, which may derive from the mineralisation of dusts settling onto the sediment surfaces of these lakes (Anderson et al. 2016), and the lower snowmelt relative to the coastal lakes limits littoral epilithic diatoms. Whilst more evidence is required to test these hypotheses, they do highlight the diverse controls on benthos throughout one discrete area of the Arctic, and suggest that a more critical interpretation of benthic:pelagic diatom ratios in sediment cores is necessary.

### Interpreting epilithic diatoms in sediment cores

**Fig. 8:**
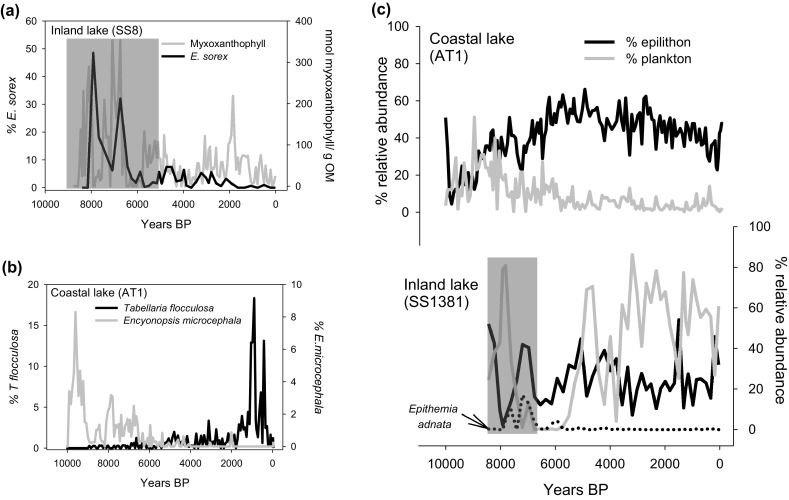
**a** Relative abundance of *Epithemia sorex* in a Holocene sediment core from Lake SS8 in the inland region (*black*) compared with the concentration of myxoxanthophyll pigment per unit organic matter sediment (*grey*). A zone of poor diatom preservation is *shaded grey*; **b** the relative abundances of *Tabellaria flocculosa* (*black*) and *Encyonopsis microcephala* (*grey*) in a sediment core from Lake AT1 in the coastal region; **c** sums of the common epilithic taxa (*black line*) as defined in Table 2 and planktonic taxa (*open circles*) in sediment cores from coastal lake AT1 and inland lake SS1381. The relative abundance of *Epithemia adnata* (*grey line*) and a zone of poor diatom preservation (*grey shaded area*) are also shown in Lake SS1381

Indicator epilithic taxa in Holocene sediment cores from across this region were able to provide new insights into previously published diatom records (Fig. 8). For example, the N_2_-fixing species *Epithemia sorex* dominated the diatom assemblage of Lake SS8 from the inland region during two periods ca. 8000 years BP and 6800 years BP (Fig. 8a). It should be noted that there are issues with diatom dissolution at this site, and so the heavily silicified *E. sorex* might be over-represented in the sediment assemblage due to preferential preservation (Law et al. 2015). However, elevated concentrations of the carotenoid myxoxanthophyll (from potentially N_2_-fixing cyanobateria) strengthen the idea that N_2_-fixers were dominant in the lake during this time period. Concurrent peaks in *Epithemia adnata* are also observed in nearby Lake SS1381, demonstrating the utility of other *Epithemia* species as indictors of nitrogen limitation (Fig. 8c). The earlier peak in inferred-nitrogen fixation around 8000BP is most likely connected to conditions when soil development was rudimentary and so inputs of N-rich DOM were limited, whereas the presence of glacially derived tills rich in P led to an imbalance in N:P ratios. However, the very dry conditions during the Holocene Thermal Maximum, which increased towards 6800BP and led to a regional drawdown of terminal lake basins (McGowan et al. 2003; Aebly and Fritz 2009) was most likely very important in limiting the hydrological transfer of nitrogen from the catchment to lake basins at this time. Such nitrogen-limited conditions are common today in lakes of arid regions (Leavitt et al. 2006), and it is possible that lake level drawdown in Lake SS8 might have provided further habitat for the colonisation of epilithic *E. sorex* species (Law et al. 2015). Epilithic indicators in Holocene records from the coastal region also provide further evidence for ontogenetic lake development in this region (Fig. 8b). For example, in lake AT1 *Encyonopsis microcephala* which strongly indicates conditions in the more alkaline and ion-rich inland/ice sheet sites is abundant in the early period of the lake development (around 9600BP). This fortifies previous interpretations of the early stages in lake ontogeny which suggests that lakes are more alkaline (Law et al. 2015), and also agrees with records of UVR-screening pigments and carotenoids (Liversidge 2012) (not shown here) which indicate that DOC and cyanobacteria were abundant in the lake, analogous to conditions in the inland lakes today. After the mid Holocene, *Tabellaria flocculosa* increased in AT1 to two maxima at 372-850BP, indicating long-term acidification of the site which was driven by the cooler and wetter conditions of the Neoglacial and associated with an increase in water clarity (Anderson et al. 2012; Liversidge 2012).

We investigated sums of epilithon in sediment records through the Holocene Thermal Maximum and Neoglacial cooling periods (Anderson et al. 2012) in coastal lake AT1 and inland lake SS1381 to investigate responses to known climatic perturbations (Fig. 8c). In Lake AT1, following on from an initial period of ontogenetic change where the pioneer species *S. pinnata* was dominant, epilithon proportions increased to a maximum around ca. 6000 BP, and declined gradually during the Neoglacial cooling period. There is, therefore, no evidence of a simple link whereby cooler conditions slow down ice melt and enhance relative epilithon growth. However, evidence from a nearby cirque basin imply enhanced cryogenic processes and catchment degradation between 5800 and 4000 BP, suggesting an increase in snowpack in this area around the time of the relative epilithon expansion (6000–4000 BP) at Lake AT1 (Anderson et al. 2012). Whilst acknowledging that controls on benthic diatoms are diverse, we infer that epilithon expansion in Lake AT1 is, in part, driven by changes in precipitation and snowpack and that the wetter period between 6000–4000 BP provided habitat for littoral diatoms which benefitted from the extended moating periods (Catalan et al. 2002). The absence of long-term patterns in epilithon relative abundance in the inland lake SS1381 where conditions are more arid and hydrological inputs from the catchment are limited suggests that such a mechanism does not operate in this lake. Therefore, interpretation of longer term patterns in epilithon abundance may be best considered alongside hydrological changes in the lake catchment. In comparison with planktonic taxa which are also strongly determined by thermal stratification characteristics in lakes (Saros et al. 2016), it might be that the linkages between epilithon and lake hydrology (and therefore climate) are more straightforward, but these hypotheses require more rigorous testing.

## Summary

We demonstrated strong correlations between water chemistry and littoral epilithic diatoms across this lake district in West Greenland, despite some spatial covariance. In particular, nitrate-nitrogen concentrations (which were greater at the coast in the spring) and silicate (more abundant in summer at the coast) were important determinants of diatom distributions. We identified that *Epithemia* species are useful indicators of nitrogen limitation via spatial surveys and long-term sedimentary sequences, and therefore, along with similar endosymbiotic taxa (*Rhopalodia* species) they might be used to infer long-term changes in lake nitrogen cycling. It proved difficult to identify early ‘pioneer’ spring taxa that could be unambiguously used as phenological indicators, although *Gomphonema* and *Hannaea arcus* were associated with spring snowmelt in some river-fed lakes at the coast. However, we identified some seasonal differences in the inland lakes where more complex biofilms developed in the summer during the longer growing season, resulting in diatom assemblages with low species richness that were small, motile and adnate. Such observations identified the potential use of *Nitzschia* spp and/or *Encyonema microcephala* as indicators of organic-rich conditions, as can occur in lakes associated with permafrost melt and catchment vegetation expansion, but preservation of these taxa in sediments is problematic. Littoral epilithic taxa have potential as palaeoecological indicators of snowmelt conditions because they are strongly influenced by nutrient delivery and chemical conditions in the spring, but interpretation of such patterns depends on the hydrological context.
